# To counteract or to clear high-mobility group box-1 protein in influenza A (H1N1) infection? That may become the question

**DOI:** 10.1186/s13054-015-1126-z

**Published:** 2015-11-23

**Authors:** Patrick M. Honore, Rita Jacobs, Inne Hendrickx, Elisabeth De Waele, Viola Van Gorp, Herbert D. Spapen

**Affiliations:** Intensive Care Unit Department, Universitair Ziekenhuis Brussel, Vrije Universiteit Brussel, 101, Laarbeeklaan, 1090 Jette (Brussels), Brussels, Belgium

Up to one third of patients with influenza A (H1N1) virus require intensive care unit (ICU) admission because of severe pneumonia, sepsis, or acute respiratory distress syndrome or a combination of these. Still, in-ICU mortality rate remains excessively high despite the deployment of maximal therapeutic support, often including extracorporeal membrane oxygenation [1].

In this perspective, the recently published experimental findings of Nosaka and colleagues in *Critical Care* may represent an important therapeutic breakthrough [2]. These investigators show that an anti-high-mobility group box-1 (anti-HMGB-1) monoclonal antibody suppresses the H1N1-induced immuno-inflammatory response, resulting in significant attenuation of lung injury and improved survival [2].

This study underscores that pharmacological inhibition of key inflammatory cytokines, despite offering equivocal or no additional benefit in sepsis, might be useful for treatment of specific infections that are accompanied by intense systemic inflammation. However, such therapy is expensive and not widely available, and little is known about eventual unwarranted side effects or late-onset complications in critically ill subjects.

Although its relatively small molecular weight does not prohibit removal by routine convective hemofiltration, HMGB-1 is effectively cleared by highly adsorptive dialysis membranes (HADMs) [3]. HADMs, in particular the surface-treated acrylonitrile 69 filter, are increasingly used for continuous renal replacement therapy (CRRT) in ICU patients [4] and may offer a valuable, more easily accessible, and potentially cheaper alternative to inhibit HMGB-1 activity in life-threatening influenza (H1N1) virus infection.

## Abbreviations

CRRT: Continuous renal replacement therapy
H1N1: influenza A
HADM: Highly adsorptive dialysis membrane
HMGB-1: High-mobility group box-1
ICU: Intensive care unit
