# Supplementary material for: To counteract or to clear high-mobility group box-1 protein in influenza A (H1N1) infection? That may become the question
Source: Crit Care. 2015 Nov 23;19:401. doi: 10.1186/s13054-015-1126-z (PMC4655500; doi:10.1186/s13054-015-1126-z)
Supplement: Related texts

# Reply: Authors’ response

**Authors:** Nobuyuki Nosaka, Masato Yashiro, Hirokazu Tsukahara, Masahiro Nishibori, Tsuneo Morishima

We thank Honore and colleagues for their helpful comments on our article on the suppressive effects of HMGB-1 monoclonal antibody against H1N1-induced pneumonia [2]. They suggest that CRRT with HADMs may be beneficial to suppress HMGB-1 activity against H1N1 infection not only with regard to effectiveness but also accessibility, safety and cost. We agree that adjunctive therapy with HADMs holds promise against severe influenza and are interested in future research investigating systemic and local HMGB-1 clearance efficiency in influenza patients; however, we would like to comment from the pediatrician’s viewpoint.

The clinical course of influenza infection is usually severe in infants, as in the 2009 pandemic. Therefore, young children should be focused on when preparing for future influenza outbreaks. Although anti-HMGB1 monoclonal antibody might be administered in cases of severe infection in general clinical settings, CRRT requires intensive care, resulting in higher medical costs. Moreover, CRRT is difficult to administer to children, especially those less than 10 kg, because of the lack of trained staff or appropriate equipment [5]. Given the estimates for the challenging demands for mechanical ventilators in a future severe pandemic [6], the feasibility of a stable supply of CRRT equipment or HADMs is questionable.

We have reported the possible therapeutic options for severe influenza pneumonia [2, 7] and believe that the preparation of a range of therapeutic choices will be crucial for future influenza outbreaks to protect the next generation.
